# Multiple inappropriate implantable cardiac defibrillator therapies in rapid succession

**DOI:** 10.1002/ccr3.1222

**Published:** 2017-10-23

**Authors:** Jeffrey A. Marbach, Colin Yeo, Martin S. Green, Girish M. Nair

**Affiliations:** ^1^ Division of Cardiology University of Ottawa Heart Institute Ottawa ON Canada

**Keywords:** Clinical, defibrillation, electrophysiology, implantable cardiac defibrillator

## Abstract

Inappropriate implantable cardiac defibrillator (ICD) shocks are associated with significant morbidity and have the potential to trigger ventricular arrhythmias, cardiac decompensation, and death. We present a case of multiple inappropriate ICD therapies in rapid succession due electromagnetic interference from a Dr‐Ho's transcutaneous electric nerve stimulator machine, and subsequently from a faulty electrical outlet.

## Case Presentation

A 74‐year‐old male with a dual chamber implantable cardiac defibrillator (ICD) presented to the emergency department (ED) after his ICD alarm started beeping. Several hours before his presentation, he was plugging his phone into an electrical socket when he felt a jolt and saw a spark, leading him to believe he had received a shock from the electrical outlet. He remained conscious throughout the episode, and on arrival to the ED, he was well with no complaints apart from his ICD alarm sounding.

ICD interrogation showed normal device and lead parameters. The ICD was programmed with a ventricular fibrillation (VF) zone to deliver burst antitachycardia pacing (ATP), followed by 35J shocks. The lead integrity alert (LIA) was triggered on the day of presentation after identifying eight nonsustained ventricular high rate events at a cycle length (CL) <220 ms.

**Figure 1:**
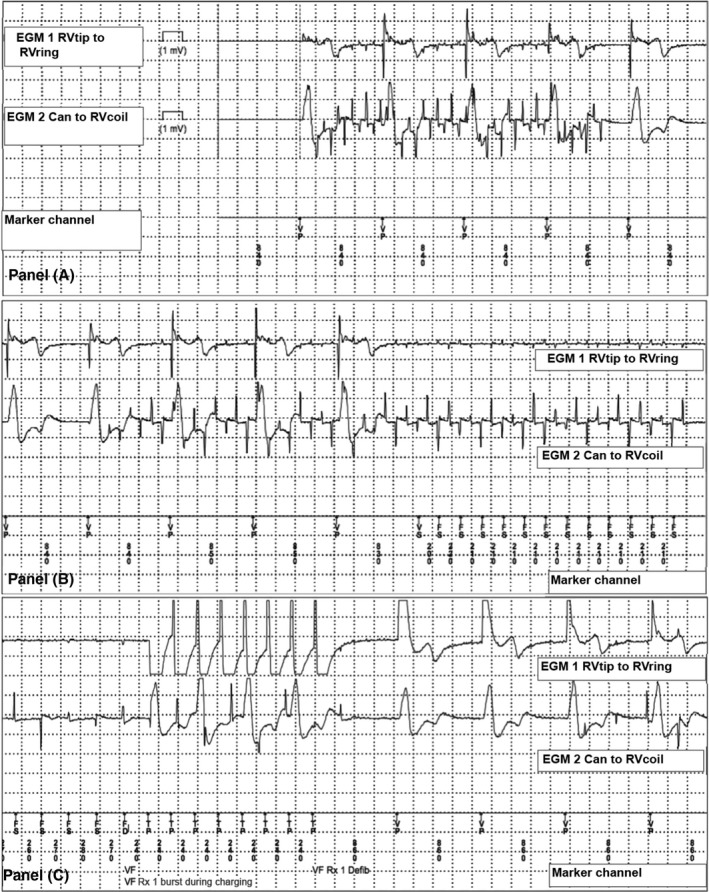
ICD tracings of near‐field (RVtip to RVring) and far‐field (Can to RVcoil) EGM during episode triggering ATP. Panel (A) ventricular pacing (VP) at a CL of 840 ms with superimposed high‐frequency deflections in both channels. Panel (B) device identifies the high‐frequency deflections as sensed activity at a CL of 210 ms (FS), which is consistent with electrical interference from a TENS machine. Panel (C) inappropriate ATP (TP) in response to the sensed TENS interference.

Minutes before the LIA was triggered, the electrogram (EGM) showed deflections in both channels (RVtip to RVring; Can to RVcoil), on a background of ventricular pacing (Fig. 1). The deflections were in the VF detection zone of 320 ms. This is followed by eight ATP deflections before the device considered the tachycardia terminated. Event log revealed six other nonsustained tachycardia episodes of the same morphology and frequency, which were short‐lived and did not result in therapies.

**Figure 2:**
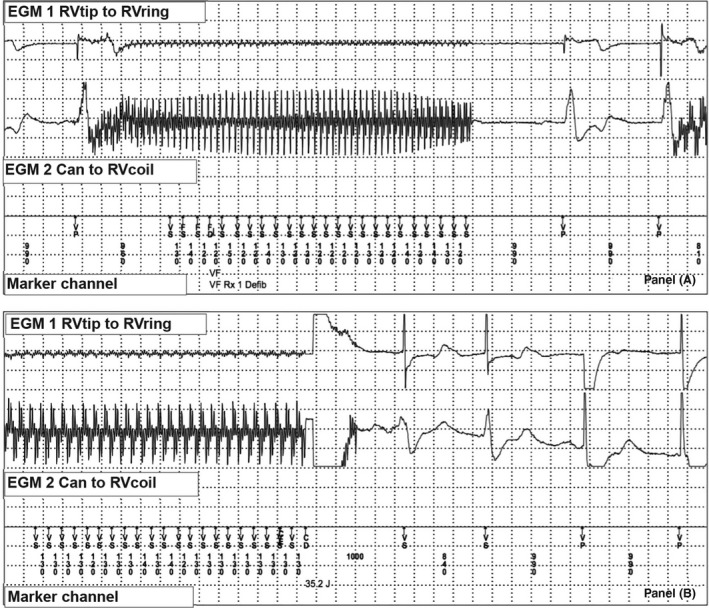
ICD tracings of near‐field (RVtip to RVring) and far‐field (Can to RVcoil) EGM during episode triggering 35J shock. Panel (A) high‐frequency deflections in both the near‐field and far‐field at a CL of 120–150 ms (VS), which is consistent with electrical interference from an AC outlet and triggers VF detection. Panel (B) inappropriate ICD shock (CD) in response to the sensed AC electrical current.

The final tachycardia event that occurred before the LIA was triggered (Fig. 2) again demonstrates deflections in both channels; however, they are of shorter CL and a different morphology than those seen in Fig. 1. As the episode's CL is very short, no ATP was performed, and the device delivered a 35J shock. No further high‐frequency deflections were detected, and the device deemed it as termination of tachycardia. Were these inappropriate therapies and if so what was the cause?

## Commentary

Figures 1 and 2 demonstrate nonphysiologic, high‐frequency intervals that are too rapid to represent ventricular arrhythmias. Therefore, we must consider that these were inappropriate therapies initiated by noncardiac signals, such as a lead fracture, a loose setscrew, or environmental sources of electrical magnetic interference (EMI).

Both events demonstrate deflections in the near‐field (RVtip to RV ring) and far‐field (Can to RVcoil) channels. Lead fracture is unlikely to present itself for the first time in both the near‐field and far‐field, as this would require extensive lead fracture. A loose setscrew may present with a nonphysiologic sensed event, but it is unlikely to surface years later. Additionally, in the setting of lead fracture or a loose setscrew we would expect to see high lead impedance on device interrogation, which was not seen in this case.

An environmental source of noise is a more likely cause of simultaneous near‐field and far‐field deflections, and therefore, a detailed history is of paramount importance. On further questioning the patient admitted to using a Dr‐Ho's transcutaneous electrical nerve stimulation (TENS)™ machine on his feet several minutes before plugging his phone into the electrical outlet. This was the first time he had used this device, and he was asymptomatic throughout use. Shortly after, he felt a jolt while he was inserting a power cord into the alternating current (AC) electrical socket. In retrospect, both of these events corresponded with the detected VF events.

In further review of the ICD tracings from the event, we see that the two separate episodes triggering therapies, although temporally related, were due to noise of different frequencies. The episode in Fig. 1 is low frequency and is consistent with EMI from a TENS, whereas in Fig. 2, we notice EMI with high‐frequency deflections (60 Hz, 16 ms interval) and alternating amplitude, which is consistent with EMI from a standard North American AC outlet. This is also consistent with the event log identifying six other nonsustained tachycardia events of similar morphology. Therefore, the EGMs and provided history suggest that these episodes were triggered by nonphysiologic noise, first from the Dr‐Ho's TENS™, and subsequently from the AC current.

Instances of inappropriate therapies have a wide variety of causes that range from intrinsic device failures (lead fracture, lead disconnections), to external electrical interference (electrocautery, electric stoves, TENS) 1. Previous reports have documented episodes of inappropriate ATP and ICD shocks from TENS therapy with both commercial machines used in chiropractic practices and with home machines 2, 3, 4, 5. One study found that TENS triggered inappropriate VT/VF detection in up to 27% of patients 6. Although manufacturers advise against the use of TENS machines in patients with pacemakers and ICDs, the risk in this population is under recognized.

This case highlights the potential for TENS machines and ungrounded electrical sockets to cause electromagnetic interference and inappropriate shocks. Uniquely, we see multiple forms of EMI occurring in the same individual in rapid succession, resulting in multiple inappropriate ICD therapies.

The risks associated with EMI in patients with an ICD remain a real problem and can lead to inappropriate device therapies, which have the potential to precipitate life‐threatening arrhythmias, further ICD therapies, and death 7. Unfortunately, patients and physicians alike are often unaware of the potential consequences of EMI. Therefore, it is essential that we provide adequate education regarding the implications of EMI and the precautions required while operating electrical equipment.

## Authorship

J. Marbach: involved in case data collection, manuscript conception and design, drafting manuscript, editing and revising manuscript, and the approval of final manuscript; C. Yeo: involved in interpreting case data, drafting manuscript, editing and revising manuscript, and the approval of final manuscript; M. Green: involved in drafting manuscript, editing and revising manuscript, and the approval of final manuscript; G. Nair: involved in manuscript conception and design, drafting manuscript, editing and revising manuscript, and the approval of final manuscript.

## Conflict of Interest

None declared.
